# Psychometric Properties of Jebsen Taylor Hand Function Test in an Italian Population with Parkinson’s Disease

**DOI:** 10.3390/healthcare12131351

**Published:** 2024-07-06

**Authors:** Giovanni Galeoto, Anna Berardi, Rachele Simeon, Francescaroberta Panuccio, Giovanni Fabbrini, Daniele Belvisi, Jerónimo González-Bernal, Jesús Ángel Seco-Calvo

**Affiliations:** 1 Department of Human Neurosciences, Sapienza University of Rome, 00185 Rome, Italy; anna.berardi@uniroma1.it (A.B.); rachele.simeon@uniroma1.it (R.S.); francescaroberta.panuccio@uniroma1.it (F.P.); giovanni.fabbrini@uniroma1.it (G.F.); daniele.belvisi@uniroma1.it (D.B.); 2 IRCSS Neuromed, Via Atinense, 18, 86077 Pozzilli, Italy; 3 Department of Nursing and Physical Therapy, Institute of Biomedicine (IBIOMED), University of León, 24071 León, Spain; jesus.seco@unileon.es; 4 Health Sciences, University of Burgos, 09001 Burgos, Spain

**Keywords:** hand dexterity, Parkinson’s disease, outcome measure, assessment

## Abstract

Background: Assessment of upper limb function is critical in the rehabilitation process of people with Parkinson’s Disease (PD), and universally validated outcome measures are needed to allow comparisons across the practice. Moreover, the study of psychometric properties of the same tool on different clinical populations guarantees the possibility of reliably evaluating the same rehabilitation treatment in people with different clinical conditions. Aim of the study: The aim of this research was to evaluate the psychometric characteristics of the Italian adaptation of the Jebsen Taylor Hand Function Test (JTHFT) in individuals with PD. Methods: The reliability and validity of the test were assessed in accordance with international standards. Internal consistency was measured using Cronbach’s alpha, and test–retest reliability was determined via the intraclass correlation coefficient (ICC). The construct validity and cross-cultural validity of the test were evaluated using Pearson’s correlation coefficient with three assessment tools on upper limb function, independence, and quality of life, with hand grip power measured by a dynamometer and an Italian pangram. Finally, responsiveness after a one month of rehabilitation treatment was measured using the Wilcoxon rank test. Results: Fifty-two Italian people with PD were recruited. Cronbach’s alpha values ranged from 0.556 (non-dominant hand) to 0.668 (dominant hand); ICC values ranged from 0.754 to 0.988. Construct validity showed that several statistically significant correlations were detected. Wilcoxon’s test showed that the assessment tool can detect a change in this population after treatment. Conclusions: The JTHFT is a reliable, valid, and respondent tool to evaluate the upper limb and hand functionalities in PD patients. It should be added to the toolkit for measuring upper limb performance in this population, adding value to clinical evaluation and ensuring comparable results for different clinical populations and different countries.

## 1. Introduction

Parkinson’s disease (PD) is a chronic degenerative disease of the central nervous system [1] and the second most common neurodegenerative disease in people over 60 [2]. The European union-wide burden for PD is estimated to be 70 disability-adjusted life years [3]. PD is primarily characterized by progressive motor symptoms [4], and these involve voluntary movements [5]. The symptomatology can, during the progression of the disease, decrease the independence of the person, contributing to a decrease of the motor function of the upper limbs and hands, with consequent functional limitation and reduction of autonomy in the activities of daily life (ADL) [6]. There is substantial evidence that motor and non-motor symptoms in individuals with PD limit their independence and social participation, resulting in a diminished quality of life (QoL) for both the patients and their caregivers [6,7]. Fluidity, coordination, effectiveness, and speed in fine and complex movements are generally reduced. This affects the ability to grasp and manipulate objects. This has an important role in altering the synchronization and integration of the components of movement as well as involving the minor amplitude of movements and the loss of regulation of the necessary force [8]. In addition, a curved posture and the reduced flexibility and use of the trunk limit functional achievement in activities [9]. It is, therefore, necessary to carry out a rehabilitation process that aims to achieve the maximum possible autonomy for the patient in different phases of the disease [10]. A correct setting for a rehabilitation program for patients with Parkinson’s also allows a careful evaluation of the functionality of the upper limbs and hands, which plays a role of particular relevance in the performance of each activity [11]. This requires a thorough assessment, which should include not only interviews and observation of occupational performance but also the use of standardized scales. Evaluating upper limb and hand function is crucial for developing an appropriate rehabilitation program, identifying limitations and residual abilities, and monitoring the progression of symptoms [12].

Over the last few decades, numerous studies have highlighted significant variability in validated tools across different national contexts [12]. While this diversity reflects the varied needs of clinical settings, it also emphasizes the necessity of adapting these tools to different contexts. Clinicians often encounter conflicting or incomplete information when making patient care decisions, exacerbated by inconsistent and non-standardized outcome assessments. This inconsistency has hindered comparative research.

To benefit patients, researchers, and clinicians, further investigation into outcome measures is needed. Universally validated outcome measures are essential for facilitating comparisons across practices. The psychometric evaluation of measurement tools for use in multiple patient populations is crucial [13]. It is now recognized that measurements used across different clinical populations must be analyzed for how they perform with varying symptoms. A scale valid in one population may not be valid in another, particularly for performance-based assessment tools that require clinician observation. Studying the psychometric properties of the same instrument in different clinical populations ensures reliable evaluation of rehabilitation treatments across diverse clinical conditions.

The scales currently used in the assessment of upper limb and hand function for Parkinson’s disease are as follows: the Unified Parkinson’s Disease Rating Scale, parts II and III (UPDRS) [14], a scale developed to evaluate various aspects of Parkinson’s disease including non-motor and motor experiences of daily living and motor complications; the Purdue Pegboard Test (PPT) [15], which measures gross movements of hands, fingers, and arms, and fingertip dexterity as necessary, in assembly tasks; the Nine-Hole Peg Test (NHPT) [16], which is used to measure finger dexterity; the Pig Tail Test (PTT) [17]; the Frenchay Arm Test (FAT), which measures upper extremity proximal motor control and dexterity during ADL performance [17]; the Action Research Arm Test (ARAT) [18], which assesses upper extremity performance (coordination, dexterity, and functioning); the Wolf Motor Function Test [19], which measures upper extremity motor ability through timed and functional tasks; the Fugl–Meyer Motor Assessment Scale, which assesses motor functioning, balance, sensation, and joint functioning the Finger-Tapping Test [17], which measures psychomotor speed; and the Jebsen and Taylor Hand Function Test (JTHFT) [20].

The Jebsen Taylor Hand Function Test (JTHFT) is a widely recognized [21,22,23,24,25,26,27,28,29,30,31] non-diagnostic assessment scale for evaluating hand and upper limb dexterity across various conditions, including muscular dystrophy [32], stroke [25,28], Parkinson’s disease [20], carpal tunnel syndrome [33], and rheumatoid arthritis [22]. This test, classified by the International Classification of Functioning, Disability, and Health (ICF) [34] as an activity assessment scale, measures changes in functional activities, making it highly advantageous for rehabilitation purposes [35]. Developed by Jebsen et al. in 1969, the JTHFT remains a cornerstone in assessing dexterity through common daily activities, such as writing and power simulation, which are quantified based on time.

Despite its long-standing use and validation in multiple languages and countries, there is a critical need to evaluate the psychometric properties of the Italian version of the JTHFT, particularly for adults with Parkinson’s disease. Given the impact of Parkinson’s disease on hand and upper limb function, having a reliable and valid tool specifically adapted and psychometrically analyzed for this population is essential [36]. While existing assessment tools for Parkinson’s disease offer valuable information on motor symptoms and overall disability, they may not fully capture the specific aspects of hand and upper limb dexterity in everyday activities. The JTHFT, with its comprehensive approach to assessing functional dexterity through real-world tasks, offers a unique advantage. However, to ensure its efficacy and reliability in the Italian-speaking Parkinson’s population, a thorough psychometric evaluation is necessary. By conducting this psychometric analysis, we aim to substantiate the relevance and applicability of the JTHFT in clinical settings, providing occupational therapists and clinicians with a robust tool tailored to the specific needs of patients with Parkinson’s disease. This will facilitate more accurate monitoring of disease progression and treatment outcomes, ultimately enhancing patient care and rehabilitation strategies.

For this reason, this study aimed to evaluate the psychometric properties of the Italian version of the JTHFT on a population of adults with Parkinson’s disease.

## 2. Methods

This study was conducted by a research group of Sapienza University of Rome RES—Rehabilitation Evidence and Development Association—who have been involved in different studies on rehabilitation [37,38,39,40,41,42,43,44,45,46].

### 2.1. Participants

The participants were enrolled at the Department of Human Neurosciences, Sapienza University of Rome, from January to August 2023. In the literature, recommendations for sample size range from 2 to 20 subjects per item [47,48]. In a systematic review of articles on sample size used for validating assessment tools, the average subject-to-item ratio was reported, with a minimum of 1 and a maximum of 527 [49]. Moreover, according to Consensus-Based Standards for the Selection of Health Status Measurement Instruments (COSMIN) checklist [50], the adequate number considered for assess internal consistency is >50 participants. Eligibility criteria for the study included a diagnosis of Parkinson’s Disease (according to the United Kingdom Parkinson’s Disease Society Brain Bank criteria) [51], the ability to understand instructions and perform the scale’s activities, and a Hoehn and Yahr (H&Y) stage between 1 and 4. The exclusion criterion was having comorbidities that affect the functionality of the upper limb. All participants were informed about the study, and their interest in participating was recorded; those who subsequently joined the study provided written consent before inclusion [52,53].

### 2.2. Clinical Assessment

The JTHFT comprises seven unilateral tasks administered using standardized procedures and verbal instructions, performed first with the non-dominant hand and then with the dominant hand. The tasks include writing a 24-letter sentence of third-grade reading difficulty; turning 3″ × 5″ (7.62 cm × 12.7 cm) cards in a simulated page-turning task; picking up small common objects such as pennies, paper clips, and bottle caps and placing them in a container; stacking checkers; simulated feeding; and moving light and heavier (1-pound) cans. The tasks are timed in seconds, with increased completion time indicating decreased hand function. A stopwatch was used for timing each task. Normative data from the original scoring system are available for both dominant and non-dominant hands.

The Health Assessment Questionnaire (HAQ), introduced in 1980, is one of the first Patient Reported Outcome (PRO) instruments designed to represent a patient-oriented outcome assessment model. The HAQ includes items that assess fine movements of the upper extremity, locomotor activities of the lower extremity, and activities involving both upper and lower extremities. Standard scoring considers the use of aids and devices or assistance from another person. It consists of 20 items in eight categories, representing a comprehensive set of functional activities—dressing, rising, eating, walking, hygiene, reach, grip, and usual activities. Each item has a four-level response set scored from 0 to 3, with higher scores indicating greater disability (0 = without any difficulty; 1 = with some difficulty; 2 = with much difficulty; and 3 = unable to do). Scores of 0 to 1 generally indicate mild to moderate difficulty, 1 to 2 indicate moderate to severe disability, and 2 to 3 indicate severe to very severe disability [54].

The Disabilities of the Arm, Shoulder, and Hand (DASH) Scale is designed to be a comprehensive instrument, assessing the upper limbs as a whole rather than limiting to a single body segment. The development of the DASH Scale was based on three theoretical domains: physical function, symptoms, and social function.

The Parkinson’s Disease Questionnaire 39 (PDQ-39), developed by Peto, was used to evaluate the change in QoL of the patient between the start of physiotherapy and the end of treatment. This scale consists of 39 items, with five answers for each question, where the worst is the fifth answer and the best is the first; the possible answers are never, occasionally, sometimes, often, and always. The scale is mainly subdivided into eight subscales: mobility (10 items), ADL (6 items), emotional well-being (6 items), stigma (4 items), social support (3 items), cognitive faculties (4 items), communications (3 items), and bodily discomfort (3 items).

Moreover, participants were assessed using an Italian pangram “Ma la volpe col suo balzo/ha raggiunto il quieto Fido”. The pangram was divided in two halves and measured by hand with a ruler to determine Area 1 and Area 2 (width × height); then, we determined the ratio between them to evaluate any progressive reduction in amplitude. The ratio is reported as percentage of Area 2 in relation with Area 1. A value of ratio less of 100% represents a reduction in amplitude, progressive micrography was set at a percentage T 30%, and a value Z 50% was assessed as severe progressive.

### 2.3. Data Analysis

The psychometric properties of the JTHFT-IT were assessed by following the COSMIN [50].

Internal consistency measures how well the items on a scale assess the same underlying concept or construct. It ensures that the items are related and collectively evaluate a single characteristic with minimal error. This property is primarily estimated using Cronbach’s alpha coefficient, which ranges from 0 to 1, with higher values indicating greater consistency. Test–retest reliability is assessed by measuring the stability of individual items when administered at different times (test–retest), with the intraclass correlation coefficient (ICC) calculated at the end. A 48 h interval was deemed appropriate for the current population, consistent with previous validation and cultural adaptation studies of the same test. According to the 95% confidence interval of the ICC estimate, values less than 0.5 indicate poor reliability, values between 0.5 and 0.75 indicate moderate reliability, values between 0.75 and 0.9 indicate good reliability, and values greater than 0.90 indicate excellent reliability [55,56].

Construct validity defines the extent to which the scores of an instrument align with hypotheses based on the assumption that the tool accurately measures the intended construct. The hypothesis tested was that the JTHFT [23] for PD is related to the power grip, handwriting skill, quality of life, and autonomy in activities of daily living. For this reason, construct validity was analyzed by comparing the scores obtained in the JTHFT with the scores obtained for power grip dynamometer force, to see the correlation between manual dexterity and strength; at the pangram, to assess the correlation between manual dexterity and writing; in the HAQ, to assess the correlation between manual dexterity and activities of daily living; in the PDQ-39, to assess the correlation between manual dexterity and quality of life; and in the DASH, which is considered as a gold standard for upper limb assessment. Construct validity assesses whether the expected relationships between constructs are observed. The following ranges were used to interpret the results: greater than 0.70 = strong correlation, between 0.50 and 0.70 = moderate correlation, and less than 0.50 = weak correlation. The significance level was set at a *p*-value of less than or equal to 0.05.

Cross-cultural validity/measurement invariance, refers to the possibility of applying a measurement instrument, initially generated in a single culture, in an equivalent way in another culture different from the original one. This property aims to investigate whether items of a tool behave similarly in different population; for this study, gender, age, age from diagnosis, Hoen and Yahr scores, motor fluctuations, and dyskinesia were considered. Mean scores and standard deviations were calculated. Moreover, box plots, showing graphical distributions of scores, were generated. Cross-cultural validity was assessed through the Pearson’s correlation coefficient (after the confirmation of the normality through the Shapiro–Wilk Test). When interpreting the results the following ranges were considered: r > 0.70 for a strong correlation; 0.50 < r < 0.70 for a moderate correlation; r < 0.50 for a weak correlation.

Responsiveness refers to an outcome measure’s ability to detect changes over time in the construct being measured. This psychometric property was measured for this study after an intervention carried out in one month (10 sessions) of both handwriting training and Occupational Therapy. The Wilcoxon rank test was used, calculating the statistical significance from the values obtained from the JTHFT at baseline and after one month of treatment.

All statistical analyses were performed using the Statistical Package for the Social Sciences (SPSS) version 20.0 for Windows. The significance level was set as a *p*-value less than or equal to 0.05 for all the psychometric properties analyzed [23].

## 3. Results

The scale was administered to 52 individuals, 69% of whom were male, with an average age of 68.75 years (standard deviation of 10.90). The demographic characteristics of the population are shown in Table 1. For reliability results, acceptable internal consistency values were obtained, with Cronbach’s alpha values ranging from 0.556 for the non-dominant hand to 0.668 for the dominant hand. Table 2 shows the mean, standard deviation, and Cronbach’s alpha values if one of the scale’s items is removed. The test–retest analysis showed good results, with ICC values between 0.754 and 0.988, demonstrating the stability of the test. The values for each Item are given in Table 3.

For validity results, Construct validity was analyzed by comparing scores obtained in the JTHFT with those obtained for the power grip dynamometer strength, pangram, HAQ, PDQ-39, and DASH; the analysis was performed in the dominant and non-dominant hand. Several statistically significant correlations were found and are shown in Table 4, Table 5 and Table 6.

Cross-cultural validity/measurement invariance was analyzed by comparing scores obtained for the JTHFT with demographic characteristics of participants; results in the dominant hand reported a statistically significant correlation between age and the first two items of Jebsen: “Writing” and “turning pages”. The results are shown in Table 7.

Finally, responsiveness was measured for this study in a subpopulation of 17 people after an intervention carried out in one month (10 sessions) of both handwriting training and occupational therapy. From the results (Table 8), it is possible to observe that the assessment tool was able to detect a change in this population for most of the items.

## 4. Discussion

This study aimed to validate the psychometric properties of the JTHFT scale in an Italian population with Parkinson’s disease. Internal consistency of the scale was assessed using Cronbach’s alpha, yielding values of 0.56 for the non-dominant hand and 0.67 for the dominant hand. Although not exceeding the minimum threshold of 0.7, it was found that by removing item 1 of the scale, i.e., the one concerning the writing of a 24-letter sentence, the value of Cronbach’s alpha increases, becoming 0.83 and exceeding the minimum threshold of 0.7, which is necessary to define the instrument as reliable. This result is in line with previous studies on the JTHFT [22,23,25]. In the Portuguese-language validation in a post-stroke population of the JTHFT, the authors attributed the increase in alpha value if the first item was deleted to the low education of the participants. This hypothesis was debunked upon the absence of statistically significant differences between the results obtained by patients with higher versus lower levels of education [25].

However, in addition to the result of this study carried out on a population with hemiparesis, it should be noted and considered that within Parkinson’s disease, among the most common symptoms are bradykinesia and a specific disorder known as micrography, which consequently lead to an increase in the time needed to carry out activities, both because the movements are somewhat slowed down and also because there is real difficulty in writing [57]. This result is also in line with the same study (on the psychometric properties of the JTHFT on a population with Parkinson’s disease) carried out in Hong Kong [20]. Indeed, comparing the administration of the JTHFT on a healthy population to a population with Parkinson’s disease, the latter appears to have needed more time to complete the activities required by the JTHFT. Also, in this case, the peculiarity of item 1 on writing emerged, even if marginally, the cause of which was explained in the same way as bradykinesia and micrography, typical of Parkinson’s. To assess the reliability of the scale, a test–retest analysis was performed by administering the test on the same person after 48 h, which showed that the instrument was stable, with ICC values between 0.754 and 0.988. This characteristic affirms that using the JTHFT in rehabilitative treatment and administering it over time would give constant results. This allows us to define it as a reliable tool for a possible follow-up. Cross-cultural validity/measurement invariance analysis showed, among all demographic characteristics, a statistically significant correlation only between participants’ age and items 1 and 2 of the JTHFT, namely writing and page rotation simulation. This is related to the fact that increasing age and symptoms of the disease, including micrography and fine dexterity, can be worsened, thus leading to an increase in the time needed to perform activities [58]. Finally, some interesting results emerged in the construct validity analysis performed with Pearson’s correlation coefficient. The overall analysis showed strong correlation between the third item of the JTHFT, “Pick up small objects”, and activities of daily living like dressing, eating, hygiene, and reach and grip measured by HAQ; the total score of this questionnaire showed a statistically significant correlation with all items of the JTHFT. In relation with QoL measured by PDQ-39, the domains of mobility and activities of daily living show statistically significant correlations with the JTHFT; these correlations are more evident for the non-dominant hand. All these subtest-related results are related to daily life activities, so they appear to be in line with what is evaluated in the JTHFT. As the time required to carry out the activities required by the JTHFT increases, difficulties in carrying out daily life activities are assessed in the HAQ. The construct validity results confirm the overall hypothesis that the JTHFT for PD is related to handwriting skill, quality of life, and autonomy in activities of daily living.

Finally, the responsiveness showed the ability of the test to detect a change in people with PD who attended a rehabilitation program; the results obtained did not show statistical significance for the second and last item.

While this study provides valuable insights into the psychometric properties of the Italian version of the Jebsen Taylor Hand Function Test (JTHFT) for patients with Parkinson’s disease, several limitations should be acknowledged. First, the sample size was relatively small. Additionally, the follow-up period to assess the responsiveness was short. Future studies should consider longer follow-up periods to better understand the stability of the JTHFT scores over time.

Addressing these limitations in future studies will be essential to confirm and extend the current findings, ensuring the Italian version of the JTHFT is a valid and reliable tool for assessing hand and upper limb dexterity in a broader Parkinson’s disease population.

## 5. Conclusions

The functioning of the upper limb is of great importance in all daily life activities. Utilizing standardized assessment tools to enhance the effectiveness of rehabilitation is, therefore, a priority in clinical practice. Despite the limitations of the study, our study provides evidence supporting the administration of the Jebsen Taylor Hand Function Test (JTHFT) in the Italian population with Parkinson’s disease (PD). The JTHFT has shown good psychometric properties internationally and in different populations. Given these encouraging results, the JTHFT appears to have potential value in the toolkit for measuring upper limb performance in this population, contributing positively to clinical evaluations.

## Figures and Tables

**Table 1:** Demographic characteristics of 52 participants with Parkinson’s disease participating in the Italian validation of Jebsen Taylor Hand Function test.

52 Participants	
Age Mean (SD)	68.75 (10.90)
Years from diagnosis	5.78 (3.59)
Motor MDS UPDRS (III)	20.192 (8.25)
Gender Frequency (%)	
Male	36 (69)
Occupation Frequency (%)	
Employee	14 (26)
Freelance	7 (14.4)
Retired	31 (59.6)
H&Y Frequency (%)	
1	9 (17.3)
2	24 (46)
3	13 (25)
4	6 (11.7)
Motor Fluctuation Frequency (%)	
NO	39 (75)
Dyskinesia Frequency (%)	
NO	38 (73)

MDS-UPDRS-III: Movement Disorder Society Unified Parkinson’s Disease Rating Scale—part III.

**Table 2:** Internal consistency: Cronbach’s alpha values of Jebsen Taylor Hand Function test in Italian people with Parkinson’s disease.

		**Mean (SD)**	**Cronbach’s Alpha If Item Deleted**
Non-Dominant Hand	Writing	55.79 (±28.08)	0.642
	Turning pages	9.62 (±5.35)	0.506
	Picking up small objects	10.43 (±3.83)	0.527
	Simulating feeding	20.10 (±17.34)	0.4521
	Stacking Checkers	9.71 (±4.1)	0.520
	Moving light cans	7.38 (±3.25)	0.515
	Moving heavy cans	6.96 (±2.91)	0.535
Total alpha non-dominant hand = 0.556			
		**Mean (±SD)**	**Cronbach’s Alpha If Item Deleted**
Dominant Hand	Writing	18.27 (±9.91)	0.748
	Turning pages	8.46 (±3.75)	0.597
	Picking up small objects	9.39 (±2.32)	0.615
	Simulating feeding	15.03 (±5.83)	0.615
	Stacking Checkers	7.95 (±3.62)	0.632
	Moving light cans	6.11 (±1.69)	0.629
	Moving heavy cans	5.85 (±1.48)	0.641
Total alpha dominant hand = 0.668			

**Table 3:** Test–retest reliability (48 h): Intraclass correlation coefficient values of Jebsen Taylor Hand Function test in Italian people with Parkinson’s disease.

		Cronbach’s Alpha If Item Deleted	Test	Retest	ICC 95% IC
			Mean (±SD)	Mean (±SD)	
Non-Dominant Hand	Writing	0.642	59.25 (±30.18)	52.29 (±24.78)	0.988 [0.96–1]
	Turning pages	0.506	10.07 (±6.27)	12.57 (±10.85)	0.986 [0.95–1]
	Picking up small objects	0.527	9.56 (±3.41)	9.44 (±3.53)	0.925 [0.72–0.98]
	Simulating feeding	0.4521	20.57 (±20.77)	16.57 (±6.82)	0.905 [0.65–0.97]
	Stacking Checkers	0.520	8.99 (±3.89)	8.22 (±4.46)	0.779 [0.18–0.94]
	Moving light cans	0.515	7.12 (±3.51)	7.00 (±3.78)	0.968 [0.88–0.99]
	Moving heavy cans	0.535	6.98 (±3.49)	7.22 (±4.25)	0.986 [0.95–1]
Dominant Hand	Writing	0.748	18.52 (±11.25)	16.91 (±4.82)	0.975 [0.91–0.99]
	Turning pages	0.597	8.57 (±4.24)	8.83 (±5.30)	0.969 [0.89–0.99]
	Picking up small objects	0.615	8.90 (±2.28)	9.71 (±3.84)	0.946 [0.8–0.99]
	Simulating feeding	0.615	13.45 (±5.16)	13.39 (±3.87)	0.960 [0.85–0.99]
	Stacking Checkers	0.632	7.51 (±3.60)	7.50 (±4.25)	0.754 [0.09–0.93]
	Moving light cans	0.629	5.97 (±1.90)	6.23 (±2.21)	0.971 [0.89–0.99]
	Moving heavy cans	0.641	5.71 (±1.67)	6.05 (±1.79)	0.952 [0.82–0.99]

**Table 4:** Construct validity: Pearson’s correlation coefficient between Jebsen Taylor Hand Function test and Pangram Area 1 (first half of the sentence) and Area 2 (second half of the sentence) in Italian people with Parkinson’s disease.

	PANGRAMM_Area 1	PANGRAMM_Area 2	PANGRAMM_Area_tot	PANGRAMM_Ratio%
Writing	0.836 **	0.817 *	0.835 **	0.395
Turning pages	0.075	0.014	0.052	0.244
Picking up small objects	0.178	0.111	0.153	0.212
Simulating feeding	0.521	0.492	0.513	0.516
Stacking Checkers	0.117	0.103	0.112	0.005
Moving light cans	−0.107	−0.192	−0.141	0.328
Moving heavy cans	−0.410	−0.474	−0.438	0.029

* = Correlation is significant at the 0.05 level (2-tailed); ** = correlation is significant at the 0.01 level (2-tailed).

**Table 5:** Construct validity: Pearson’s correlation coefficient between Jebsen Taylor Hand Function test and Health Assessment Questionnaire (HAQ), the Disabilities of the Arm, Shoulder, and Hand (DASH), and the Dynamometer in Italian people with Parkinson’s disease.

JTHFT	Health Assessment Questionnaire									DASH	Dynamometer	
	Dressing	Arising	Eating	Walking	Hygiene	Reach	Grip	Activity	Total		ND	D
1 ND	0.15	0.16	0.28	0.391 *	0.27	0.19	0.17	0.31	0.21	0.05	−0.16	−0.26
1 D	0.28	0.07	0.377 *	0.420 *	0.22	0.437 *	0.30	0.29	0.36	−0.06	−0.05	−0.01
2 ND	0.36	0.31	0.28	0.665 **	0.25	0.485 **	−0.12	0.500 **	0.413 *	0.21	−0.728 **	−0.880 **
2 D	0.29	0.25	0.25	0.522 **	0.05	0.29	0.07	0.386 *	0.33	0.21	−0.658 *	−0.710 **
3 ND	0.469 *	0.447 *	0.31	0.643 **	0.462 *	0.626 **	0.05	0.550 **	0.545 **	0.51	−0.757 **	−0.788 **
3 D	0.442 *	0.375 *	0.09	0.403 *	0.33	0.29	0.11	0.521 **	0.399 *	0.628 *	−0.710 **	−0.686 **
4 ND	0.06	−0.02	0.36	0.421 *	0.01	0.26	0.18	0.06	0.20	−0.22	0.00	−0.01
4 D	0.08	0.06	0.14	0.23	−0.06	0.00	0.16	0.19	0.11	−0.09	−0.52	−0.48
5 ND	0.389 *	0.28	0.32	0.600 **	0.450 *	0.668 **	0.16	0.414 *	0.463 *	0.34	−0.541 *	−0.44
5 D	0.07	−0.01	−0.12	0.11	0.08	0.22	0.14	0.26	0.11	0.12	−0.37	−0.32
6 ND	0.27	0.35	0.26	0.723 **	0.22	0.411 *	0.04	0.430 *	0.409 *	0.21	−0.667 **	−0.622 *
6 D	0.33	0.27	0.21	0.33	0.22	0.20	0.22	0.437 *	0.34	−0.02	−0.620 *	−0.564 *
7 ND	0.462 *	0.382 *	0.24	0.615 **	0.375 *	0.541 **	0.05	0.554 **	0.500 **	−0.01	−0.763 **	−0.725 **
7 D	0.470 *	0.32	0.24	0.37	0.450 *	0.438 *	0.35	0.560 **	0.503 **	−0.41	−0.583 *	−0.44

JTHFT = Jebsen Taylor Hand Function Test; ND = Non-Dominant Hand; D = Dominant Hand; DASH = Disability Arm Shoulder and Hand. 1 = Writing; 2 = Turning pages; 3 = Picking up small objects; 4 = Simulating feeding; 5 = Stacking Checkers; 6 = Moving light cans; 7 = Moving heavy cans. * = Correlation is significant at the 0.05 level (2-tailed); ** = correlation is significant at the 0.01 level (2-tailed).

**Table 6:** Construct validity: Pearson’s correlation coefficient between Jebsen Taylor Hand Function test and the Parkinsons’ Disease Questionnaire (PDQ-39) in Italian people with Parkinson’s disease.

	Parkinson’s Disease Questionnaire-39							
	Mobility	Activities of Daily Living	Emotional Well-Being	Stigma	Social Support	Cognition	Communication	Bodily Discomfort
Writing (ND)	0.232	0.038	0.148	−0.163	−0.077	−0.096	−0.051	−0.134
Writing (D)	0.215	0.112	0.157	−0.197	−0.002	0.275	−0.101	−0.030
Turning pages (ND)	0.483 **	0.378 *	0.124	0.069	−0.144	0.192	0.074	0.202
Turning pages (D)	0.245	0.209	−0.055	0.097	−0.061	0.056	−0.039	−0.051
Picking up small objects (ND)	0.408 **	0.349 *	0.265	0.027	−0.123	0.199	0.064	0.267
Picking up small objects (D)	0.154	0.315	−0.080	0.105	−0.121	−0.042	−0.150	0.138
Simulating feeding (ND)	0.115	−0.005	0.091	−0.130	−0.119	0.134	−0.135	−0.048
Simulateing feeding (D)	−0.003	0.039	−0.074	0.273	−0.167	−0.125	−0.107	0.009
Stacking Checkers (ND)	0.406 *	0.357 *	0.314	0.073	−0.150	0.337 *	−0.006	0.261
Stacking Checkers (D)	0.029	0.107	−0.108	−0.026	−0.147	0.073	−0.155	0.066
Moving light cans (ND)	0.368 *	0.290	0.169	−0.015	−0.249	0.229	−0.136	0.321 *
Moving light cans (D)	0.101	0.302	−0.041	0.169	−0.248	−0.194	−0.091	0.020
Moving heavy cans (ND)	0.473 **	0.462 **	0.293	0.092	−0.136	0.340 *	0.039	0.359 *
Moving heavy cans (D)	0.282	0.455 **	0.213	0.046	−0.077	0.086	0.125	0.220

JTHFT = Jebsen Taylor Hand Function Test; ND = Non-Dominant Hand; D = Dominant Hand. 1 = Writing; 2 = Turning pages; 3 = Picking up small objects; 4 = Simulating feeding; 5 = Stacking Checkers; 6 = Moving light cans; 7 = Moving heavy cans. * = Correlation is significant at the 0.05 level (2-tailed); ** = correlation is significant at the 0.01 level (2-tailed).

**Table 7:** Cross-cultural validity/measurement invariance: Pearson’s correlation coefficient between Jebsen Taylor Hand Function Test and demographic characteristics of the 52 participants with Parkinson’s disease participating in the study.

	Age	Gender	Age from Diagnosis	Hoehn and Yahr	Motor Fluctuations	Dyskinesia
Writing	0.557 **	0.092	0.267	0.183	0.105	0.217
Turning pages	0.444 *	−0.031	−0.006	0.306	−0.034	0.155
Picking up small objects	0.338	0.285	0.033	0.037	0.091	0.182
Simulating feeding	0.185	0.109	−0.009	0.040	0.197	0.320
Stacking Checkers	0.285	0.139	−0.169	−0.214	−0.038	0.041
Moving light cans	0.298	0.244	−0.079	−0.071	0.175	0.319
Moving heavy cans	0.215	0.298	0.088	0.067	0.121	0.328

* = Correlation is significant at the 0.05 level (2-tailed); ** = correlation is significant at the 0.01 level (2-tailed).

**Table 8:** Responsiveness: Wilcoxon rank test on values obtained from the JTHFT at baseline and after one month of treatment (10 sessions).

	T0—Baseline		T1—One Month		Z	*p*
Writing (ND)	50.20 (27.9)	41.66	42.64 (14.3)	42.17	−0.178	0.86
Writing (D)	17.75 (6.7)	15.57	14.92 (3)	13.65	−1.778	0.08 *
Turning pages (ND)	8.69 (2.5)	7.60	7.00 (1.2)	6.88	−1.334	0.18
Turning pages (D)	8.23 (2.6)	7.17	7.00 (2)	6.68	−0.489	0.62
Picking up small objects (ND)	12.23 (4.2)	10.37	11.61 (4.4)	9.98	−2.045	0.04 *
Picking up small objects (D)	10.40 (2.2)	10.37	9.30 (1.8)	9.61	−2.118	0.03 *
Simulating feeding (ND)	19.12 (6.4)	18.60	19.49 (4.4)	18.89	−2.401	0.02 *
Simulating feeding (D)	18.31 (6)	18.35	15.65 (2.9)	15.38	−1.778	0.08
Stacking Checkers (ND)	11.22 (4.3)	9.66	9.68 (3.4)	8.55	−2.045	0.04 *
Stacking Checkers (D)	8.87 (3.7)	8.58	7.69 (3.4)	7.20	−1.689	0.09
Moving light cans (ND)	7.92 (2.7)	7.19	6.60 (1)	6.78	−2.223	0.03 *
Moving light cans (D)	6.38 (1.2)	6.41	6.20 (1.2)	6.57	−2.578	0.01 *
Moving heavy cans (ND)	6.91 (1.1)	6.79	6.07 (1.1)	5.85	−0.623	0.53
Moving heavy cans (D)	6.14 (1)	6.08	5.88 (1.2)	6.12	−1.478	0.14

* = Correlation is significant at the 0.05 level (2-tailed).

## Data Availability

The datasets generated and/or analyzed during the current study are available from the corresponding author on reasonable request.
